# Feasibility of transthoracic echocardiography in the prone position with a low-cost adaptation: morphological, functional, and hemodynamic analysis

**DOI:** 10.31744/einstein_journal/2026AO1802

**Published:** 2026-03-27

**Authors:** Nilson Bossle Conci, Nathalia Conci Santorio, Pandreli Testa Santorio, Juliana Cassiano Lemos, Fábio Fernandes, Viviane Tiemi Hotta

**Affiliations:** 1 Universidade de São Paulo Faculdade de Medicina São Paulo SP Brazil Faculdade de Medicina, Universidade de São Paulo, São Paulo, SP, Brazil.; 2 Cachoeiro de Itapemirim Unimed Sul Capixaba ES Brazil Unimed Sul Capixaba, Cachoeiro de Itapemirim, ES, Brazil.; 3 Universidade de São Paulo Instituto do Coração Faculdade de Medicina São Paulo SP Brazil Instituto do Coração (InCor), Faculdade de Medicina, Universidade de São Paulo, São Paulo, SP, Brazil.; 4 Fleury Medicina e Saúde São Paulo SP Brazil Fleury Medicina e Saúde, São Paulo, SP, Brazil.

**Keywords:** Prone position, Echocardiography, transthoracic, Hemodynamics, Point-of-care systems, Equipment design

## Abstract

**Objective:**

Transthoracic echocardiography is essential for hemodynamic assessment in critically ill patients; however, its use in the prone position remains challenging. This study aimed to assess the feasibility of performing transthoracic echocardiography using a low-cost ultrasound table adaptation in the prone position.

**Methods:**

This was a single-center, cross-sectional, comparative pilot study. Anthropometric and clinical data were collected along with echocardiographic image acquisition in the conventional and prone positions by the same operator. Feasibility was assessed by the acquisition of echocardiographic windows and the number of key variables in both positions. Comparative analyses were conducted for image quality, examination duration, and quantitative parameters such as variables related to systemic congestion, biventricular function, and cardiac output estimation. Statistical analysis was performed using the Wilcoxon signed-rank test, Fisher's exact test, binomial test, and false discovery rate correction for multiple comparisons.

**Results:**

Echocardiographic views were obtained for all patients in both positions. Imaging in the prone position allowed consistent acquisition of 16 of 17 key variables, with no significant difference. Image quality in the parasternal view was slightly reduced in the prone position but remained appropriate (p=0.015). Examinations in the prone position were on average 4.2 min longer (p=0.037). No significant differences were observed between the positions for any quantitative echocardiographic parameters, including chamber dimensions, systolic and diastolic function, cardiac output, and inferior vena cava assessment.

**Conclusion:**

Transthoracic echocardiography in the prone position was feasible, with measurements comparable to those in the conventional position in healthy individuals undergoing routine evaluations in a regular echocardiography laboratory. Further studies are required to validate the accuracy and applicability of this method, particularly for patients in intensive care and emergency settings.

## Highlights

All standard echocardiographic views were obtained in the prone position.

In the prone position, 16 of 17 key variables were consistently acquired.

Image quality was slightly reduced only in the parasternal view.

Quantitative measurements were comparable between the prone and supine positions.

## In Brief

Transthoracic echocardiography in the prone position using a low-cost table adaptation proved feasible, allowing for consistent acquisition of standard views and key variables. Image quality and quantitative measurements were comparable to those of conventional supine examination, supporting its potential use in critical care.

## Graphical abstract

**Figure:**
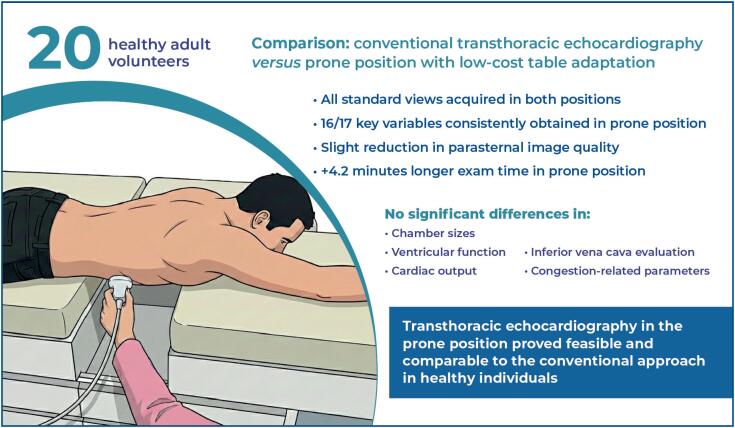

## INTRODUCTION

Transthoracic echocardiography (TTE) is becoming increasingly essential in the diagnosis and management of critically ill patients, either as a comprehensive examination or a targeted tool for obtaining specific clinical information. Various parameters can be rapidly assessed, such as biventricular function, dimensions, and respiratory variability of the inferior vena cava, presence of pericardial effusion, transvalvular flows, estimation of cardiac output, and pressure in the left atrium and pulmonary artery. Integrating these findings with the clinical presentation allows for improved assessment of different shock etiologies and aids in fluid management, vasoactive therapy, and mechanical ventilation parameters.^(1)^

Acute respiratory distress syndrome (ARDS) is an extreme form of hypoxemic acute respiratory failure associated with multiple etiologies, such as pneumonia (mainly bacterial and viral), gastric content aspiration, sepsis from other sources, non-cardiogenic shock, pancreatitis, severe trauma, and major surgeries.^(2)^ According to the Berlin criteria for ARDS,^(3)^ it is essential to rule out primary cardiac causes. In this context, TTE is a highly useful tool for clinicians owing to its availability, speed, and relatively low cost.

Given the high incidence of severe hypoxemia, patients with ARDS are often placed in the prone position, as this posture enhances ventilation in the dependent alveolar regions of the lung bases. However, prone positioning limits the acquisition of conventional echocardiographic windows. Recently, owing to the coronavirus disease 2019 (COVID-19) pandemic, the number of patients with ARDS ventilated in the prone position has significantly increased.

Several studies have evaluated the use of TTE in the prone position, either with a transesophageal transducer^(4,5)^ or a standard transducer^(6-14)^ (Table 1). When a standard transducer is used, techniques such as the swimmer's position with elevation of the left upper limb,^(6,7)^ partial deflation of the pneumatic mattress,^(8)^ and the placement of a pillow under the left upper limb to elevate the chest have been described.^(10-13)^ However, in most of these studies, the parasternal window could not be obtained using a transthoracic transducer, which is an important limiting factor in performing the examination.

**Table 1:** Comparison between studies that evaluated the use of transthoracic echocardiography in the prone position

Author	N	Strategy used	Obtained windows
Gibson et al., 2020^(9)^	27	"Swimmer position" with a pillow	Apical and right intercostal
García-Cruz et al., 2020^(10)^	15	"Swimmer position" with a pillow	Apical
Santos-Martínez et al., 2020^(11)^	50	"Swimmer position" with a pillow	Apical
Roemer et al., 2020^(12)^	24	"Swimmer position" with a pillow	Apical and right intercostal
Giustiniano et al., 2020^(8)^	8	Partial deflation of the pneumatic mattress	Apical
Marvaki et al., 2021^(5)^	21	Transesophageal transducer	Apical, subcostal, and parasternal
Taha et al., 2021^(4)^	30	Transesophageal transducer	Parasternal
Ugalde et al., 2022^(6)^	68	"Swimmer position"	Apical
Cheong et al., 2022^(7)^	35	"Swimmer position"	Apical
Nuzhny et al., 2023^(14)^	14	Special medical bed with an access hole for heart examination	Apical and parasternal
Del Castillo et al., 2023^(13)^	43	"Swimmer position" with pillow	Apical and subcostal

More recently, in 2023, Nuzhny et al. performed cardiac examinations on 14 healthy male participants using an adapted medical bed with an access opening, which enabled the acquisition of parasternal windows.^(14)^ Their study focused on evaluating hemodynamic variables but did not include a comparative analysis of cardiac cavity dimensions and biventricular function parameters in the prone and conventional positions.

## OBJECTIVE

This study aimed to assess the feasibility of Transthoracic echocardiography in the prone position using a more accessible adaptation that expands the range of windows for analysis and enhances image acquisition.

## METHODS

Study population and setting

This single-center, cross-sectional, comparative pilot study was conducted at one of the authors’ private clinics in Mimoso do Sul, ES, Brazil. This study was approved by the Research Ethics Committee of *Unimed Vitória* (CAAE: 57816122.2.0000.5061; #6.276.364). All TTE examinations were performed by the same operator (NBC) between August and November 2023.

The sample was selected using a convenience sampling method, and healthy individuals were invited to participate voluntarily in the study. All participants understood the procedures, voluntarily agreed to participate, and signed an informed consent form in accordance with the Good Clinical Practice guidelines and national regulations.

Anthropometric and clinical data (sex, age, noninvasive blood pressure, weight, and height) were collected. Weight and height were measured using a Welmy R-110^®^ mechanical anthropometric scale. Blood pressure was measured using the Premium^®^ aneroid sphygmomanometer. Both devices were tested and calibrated before use. Weight and height measurements were used to calculate body mass index (BMI) and body surface area using the method described by DuBois.^(15)^

### Echocardiographic assessment

**Figure 1:**
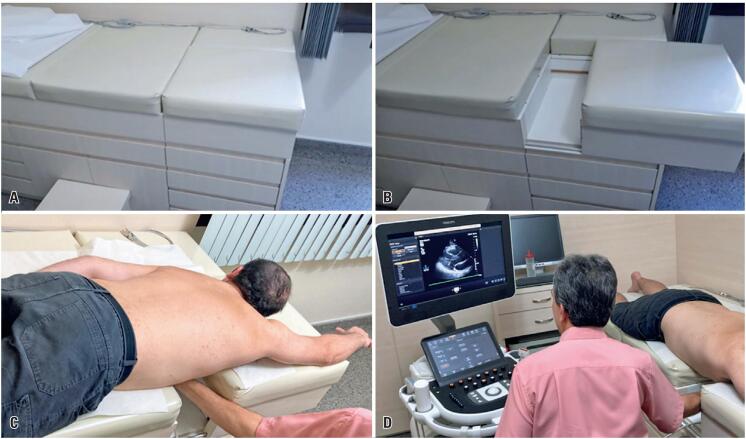
Adapted ultrasound table with an opening to allow access to the precordial region. (A) Table closed. (B) Table open. (C) Patient in the prone swimmer's position, with the right upper limb elevated. (D) Examiner's position for image acquisition

Echocardiographic image acquisition was performed using the EKO 7^®^ echocardiography device (Samsung-Medison, Hongcheon-gun, South Korea) and the sectorial transducer P2-4 BA (2-4 MHz), according to the following sequence. Initially, the inferior vena cava was assessed in the subcostal view with the patient in the supine position, while the remaining measurements were obtained in the left lateral semi-decubitus position, using apical four- and two-chamber views, as well as parasternal long- and short-axis views. Thereafter, the same views were obtained in the prone position with the patient in a swimmer's position and the right upper limb elevated using a specially adapted ultrasound table with an opening to allow access to the precordial region (Figure 1).

Adaptation developed by the first author.

Images corresponding to four cardiac cycles were acquired at both positions for quantitative analysis. Linear echocardiographic measurements were performed, including the left ventricular diastolic and systolic diameters, anteroposterior diameter of the left atrium, diameters of the sinus of Valsalva, proximal ascending aorta, left ventricular outflow tract, interventricular septum, and posterior wall of the left ventricle. Diastolic function measurements were also obtained, including the E wave of mitral inflow, E/A ratio of mitral inflow, medial e’, lateral e’, E/e’ ratio, and left atrial indexed volume. Additionally, parameters associated with hemodynamic variables and biventricular function were assessed, such as the velocity-time integral (VTI) of the left ventricular outflow tract using pulsed-wave Doppler, left ventricular ejection fraction (LVEF) using the Simpson and Teichholz methods, tricuspid annular plane systolic excursion (TAPSE), inferior vena cava (IVC) diameter during normal and deep respiration, and peak velocity of tricuspid regurgitation, if present.

The duration of the examination in each position was recorded based on the time from the start to the end of image acquisition. The operator performing the echocardiograms was unaware that the duration of the examinations would be analyzed, thereby ensuring that this information did not influence image acquisition. Image quality was subsequently assessed by an independent experienced echocardiographer who was blinded to the patient's position and identity. To minimize bias, the images were randomized before analysis and classified as poor, fair, good, or excellent.

Furthermore, the number of successfully acquired variables per acoustic window in each position was documented as an indicator of feasibility based exclusively on primary measurements, rather than values derived from subsequent calculations. Seventeen variables were considered: eight obtained from the apical window, eight from the parasternal window, and one from the subcostal window. For the apical window, these variables included: E wave, E/A ratio, medial and lateral e’, left atrial volume, left ventricular outflow tract VTI, LVEF using Simpson's method, and TAPSE. For the parasternal window, the following measurements were included: the diameter of the sinuses of Valsalva, ascending aorta diameter, anteroposterior diameter of the left atrium, left ventricular end-diastolic and end-systolic diameters, interventricular septum thickness, and posterior wall thickness. Finally, for the subcostal window, the diameter of the IVC was considered.

Additionally, the feasibility of acquiring variables potentially relevant to the clinical management of patients with ARDS was analyzed separately. These included cardiac output estimation, assessment of left ventricular function using Simpson's or Teichholz methods, right ventricular function using TAPSE, estimation of pulmonary artery systolic pressure (PASP), assessment of IVC dimensions, and collapsibility.

All parameters were obtained following the recommendations of the American Society of Echocardiography and European Association of Cardiovascular Imaging.^(16)^ Volumetric measurement of the left atrium was performed using the area-length method with apical two- and four-chamber views. Cardiac output was calculated by estimating the stroke volume through the VTI of the left ventricular outflow tract multiplied by the patient's heart rate during the examination. Pulmonary capillary wedge pressure (PCWP) was estimated from the E/e’ ratio, as described by Nagueh et al.^(17)^ Left ventricular linear measurements were used to estimate left ventricular mass, following the formula proposed by Devereux et al.^(18)^ and indexed to body surface area.

### Statistical analysis

Variables were described using the mean, standard deviation, median, and first and third quartiles. Comparisons between the prone and supine positions were performed using the non-parametric Wilcoxon signed-rank test^(19)^ with two-sided p-values. Additional analyses included Fisher's exact test, binomial test, and correction for multiple comparisons using the Benjamini-Hochberg false discovery rate procedure when appropriate. All analyses were conducted by an independent statistician using the R statistical software (R Core Team, 2024).^(20)^

## RESULTS

The study included 20 consecutive participants, half of whom were male, aged between 21 and 59 years. No participants were excluded because of acoustic window limitations, and none had chest deformities or breast implants. The clinical characteristics of the participants are presented in table 2.

**Table 2:** Patient characteristics

Variable	Mean (standard deviation)	Median [Q1; Q3]
Age (years)	39.5 (12.9)	38.0 [29.0; 52.0]
BMI (kg/m²)	26.4 (2.96)	25.4 [24.6; 27.3]
Body surface area (m²)	1.80 (0.18)	1.81 [1.73; 1.88]
SBP (mmHg)	116 (10.6)	115 [110; 120]
DBP (mmHg)	73.5 (6.71)	70.0 [70.0; 80.0]

BMI: body mass index; SBP: systolic blood pressure; DBP: diastolic blood pressure.

All echocardiographic windows were successfully obtained in 100% of participants in both positions. Table 3 summarizes the findings related to technical feasibility and image acquisition quality in the prone and conventional positions.

**Table 3:** Parameters associated with the feasibility and quality of echocardiographic image acquisition in the prone and conventional positions

Variable		Prone position	Conventional position	p value
Number of successfully acquired variables per window per patient (median, [Q1; Q3])				
	Parasternal long-axis (n=8)	8.00 [6.75; 8.00]	8.00 [8.00; 8.00]	0.412
	Apical (n=8)	8.00 [7.00; 8.00]	8.00 [8.00; 8.00]	
	Subcostal (n=1)	1.00 [1.00; 1.00]	1.00 [1.00; 1.00]	
	Total (n=17)	16.0 [15.0; 17.0]	17.0 [17.0; 17.0]	
Variables of clinical relevance for ARDS management, n (%)				
	Cardiac output estimation	19 (95)	20 (100)	***
	Assessment of LVEF (Simpson or Teichholz methods)	20 (100)	20 (100)	***
	TAPSE	17 (85)	20 (100)	0.233
	PASP	2 (10)	5 (25%)	0.408
	IVC assessment	20 (100)	20 (100)	***
Duration of the echocardiographic exam, min (median, [Q1; Q3])*		19.0 [17.7; 22.1]	14.8 [13.9; 17.5]	0.037*
Image quality scoring in parasternal long-axis window**, n (%)				
	Poor	0 (0)	0 (0)	0.015*
	Fair	1 (10)	0 (0)	
	Good	8 (80)	7 (70)	
	Excellent	1 (10)	3 (30)	
Image quality scoring in apical four-chamber window**, n (%)				
	Poor	1 (10)	0 (0)	0.345
	Fair	4 (40)	1 (10)	
	Good	3 (30)	3 (30)	
	Excellent	2 (20)	6 (60)	

* Significant p-value

** Data available for 10 patients.

*** Statistical test not applicable due to absence or minimal variation in the data.

TAPSE: tricuspid annular plane systolic excursion; PASP: pulmonary artery systolic pressure; IVC: inferior vena cava; LVEF: left ventricular ejection fraction.

The median number of successfully acquired variables was slightly lower in the prone position (16.0) than that in the conventional position (17.0); however, the difference was not statistically significant (p=0.412). No statistically significant differences were found between the positions for variables considered clinically relevant for ARDS.

The duration of the examination was significantly longer in the prone position (median, 19.0 min) than in the conventional position (median, 14.8 min; p = 0.037). No patient reported discomfort during the examination, and early termination was not requested by any participant.

Image quality assessment revealed a significant difference in the parasternal window (p=0.015), mainly due to a higher frequency of "excellent" scores in the conventional position (30% *versus* 10%) and a higher frequency of "good" scores in the prone position (80% *versus* 70%). No significant difference was observed in the apical window between the two positions. Examination duration and image quality were assessed in 10 participants.

Clinically relevant variables for ARDS management were obtained with reasonable success rates, except for PASP, which was estimated based on the peak velocity of tricuspid regurgitation. This measurement was feasible in only 10% of the patients in the prone position and in 25% in the conventional position. The primary reason for not obtaining measurements was the absence of tricuspid regurgitation. Additionally, no measurements were limited by the limitations of the acoustic window.

**Figure 2:**
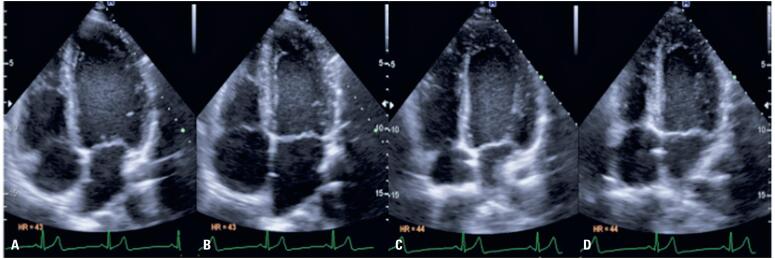
Comparison of apical four-chamber images between the conventional and prone positions. (A, B) Images obtained with the patient in the conventional position. (C, D) Images obtained with the patient in the prone position. Images A and C were obtained during diastole, and images B and D during ventricular systole

**Figure 3:**
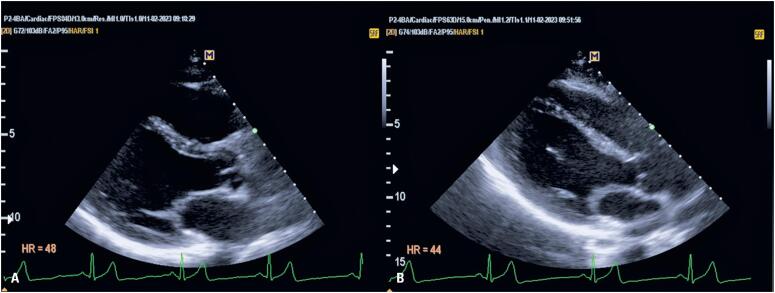
Comparison of parasternal long-axis images between the conventional position (A) and prone position (B)

Figures 2 and 3 show a comparison of the images obtained at different positions in the same patient.

Tables 4 and 5 present a quantitative comparison of the two methods. After correcting for multiple comparisons, no statistically significant differences were observed between the prone and conventional positions for any of the echocardiographic parameters analyzed.

**Table 4:** Comparison of linear echocardiographic measurements between the positions

Variable	N	Prone position		Conventional position		p value
		Mean (S.D.)	Median [Q1;Q3]	Mean (S.D.)	Median [Q1;Q3]	
Sinus of Valsava, mm	20	28.2 (3.38)	28.2 [26.2;30.3]	28.1 (3.58)	28.3 [26.1;29.8]	0.840
Proximal ascending aorta, mm	18	27.5 (4.07)	27.3 [25.9;29.8]	25.6 (2.63)	26.1 [24.2;27.2]	0.296
Anteroposterior diameter of the left atrium, mm	17	33.2 (3.17)	32.8 [31.3;33.9]	35.2 (4.28)	35.1 [31.1;38.4]	0.181
Left ventricular outflow tract, mm	20	21.0 (1.86)	20.7 [19.6;22.3]	20.8 (1.83)	20.3 [19.6;21.9]	0.655
Interventricular septum, mm	20	8.18 (1.29)	8.20 [7.16;9.20]	8.48 (1.55)	8.30 [7.56;8.90]	0.655
Left ventricular diastolic diameter, mm	20	45.5 (3.48)	46.5 [43.8;47.4]	47.1 (4.31)	48.0 [45.9;49.1]	0.113
Left ventricular posterior wall, mm	20	8.56 (0.74)	8.52 [8.05;9.03]	8.35 (1.17)	8.40 [7.77;9.23]	0.655
Left ventricular systolic diameter, mm	20	28.8 (4.50)	28.9 [25.5;32.0]	29.7 (3.56)	28.7 [28.1;31.9]	0.428
Left ventricular mass index (g/m^2^)	20	101 (13.5)	101 [98.0;105]	110 (17.8)	107 [98.4;125]	0.113

SD: standard deviation.

**Table 5:** Comparison of diastolic function measurements, hemodynamic variables, and biventricular function parameters between the positions

Variable	N	Prone position		Conventional position		p value
		Mean (S.D.)	Median [Q1;Q3]	Mean (S.D.)	Median [Q1;Q3]	
E wave of mitral inflow, mm/s	19	82.3 (15.6)	81.0 [71.0;92.5]	82.4 (12.0)	84.0 [74.0;92.5]	0.840
E/A ratio of mitral inflow	19	1.81 (0,74)	1.69 [1.42;2.26]	1.64 (0.69)	1.49 [1.17;1.91]	0.133
Medial e’, mm/s	19	12.9 (3.84)	14.0 [9.25;15.8]	12.8 (2.97)	13.0 [11.5;14.5]	0.840
Lateral e’, mm/s	19	16.0 (3.31)	16.0 [13.2;17.8]	16.6 (3.39)	17.0 [14.0;18.5]	0.655
Average e’, mm/s	19	14.5 (3.38)	14.5 [12.0;16.5]	14.7 (2.60)	15.0 [13.2;16.5]	0.655
E / e’ ratio	18	5.73 (1.17)	5.88 [4.85;6.62]	5.37 (1.67)	5.52 [4.84;6.12]	0.655
Left atrial volume index, mL/m²	17	25.9 (5.06)	24.9 [23.1;28.3]	28.5 (6.13)	29.0 [24.8;30.6]	0.372
VTI of left ventricular outflow tract, cm	19	19.9 (3.11)	19.0 [17.9;21.9]	21.1 (3.14)	21.4 [18.5;22.9]	0.372
Systemic stroke volume, mL	19	69.2 (17.5)	65.7 [58.7;73.4]	72.7 (19.0)	71.6 [57.6;83.4]	0.655
Heart rate, bpm	20	62.7 (14.0)	60.0 [53.8;74.0]	65.6 (14.4)	63.5 [55.8;73.2]	0.275
Systemic cardiac output, ml/min	19	4190 (1304)	3943 [3303;4350]	4672 (1211)	4495 [3826;5384]	0.275
Pulmonary capillary wedge pressure, mmHg	18	9.00 (1.45)	9.19 [7.91;10.1]	8.92 (1.34)	8.80 [8.18;9.62]	0.764
LVEF, Teichholz, %	20	65.7 (10.1)	64.3 [56.6;73.4]	66.4 (7.19)	68.1 [59.7;72.8]	0.797
LVEF, Simpson, %	19	65.4 (9.60)	65.6 [56.3;72.8]	63.9 (5.92)	64.6 [59.9;66.9]	0.775
TAPSE, mm	17	24.1 (4.71)	23.5 [21.3;24.8]	25.3 (4.99)	25.3 [22.4;29.0]	0.655
Inferior vena cava, mm	20	16.5 (4.20)	18.0 [12.2;20.2]	15.4 (3.97)	14.9 [12.3;19.5]	0.655

SD: standard deviation; VTI: velocity-time integral; LVEF: left ventricular ejection fraction; TAPSE: tricuspid annular plane systolic excursion.

## DISCUSSION

Our findings demonstrated that TTE was feasible in the prone position with adequate visualization of the apical, parasternal, and subcostal windows after low-cost adaptation. No statistically significant difference was observed in the number of variables successfully acquired in the prone and conventional positions.

A 2008 study reported that the prone position is associated with anterior displacement of the superior and lateral portions of the heart, as well as a reduction in lung volume between the heart and chest wall.^(21)^ In images obtained from the parasternal long-axis window in the prone position, the cardiac apex was laterally and superiorly displaced, making the longest cardiac axis more parallel to the intercostal spaces. This orientation allowed for a wider view of the apical region in the parasternal long-axis window, which is uncommon in examinations performed in the conventional position. Moreover, the proximity between the heart and chest wall may improve the acoustic window in selected patients, such as those with obesity or have lung diseases, potentially providing a benefit even in an outpatient setting.

Additionally, no differences were observed in clinically relevant variables for ARDS management, such as cardiac output estimation, LVEF assessment, TAPSE, and IVC assessment. These measurements were successfully obtained in the prone position for a large proportion of the participants (95%, 100%, 85%, and 100%, respectively).

Compared to other studies that used a transthoracic transducer without ultrasound table adaptation,^(6-13)^ we obtained similar success rates for the acquisition of biventricular function parameters in all participants, with higher success in obtaining parasternal long- and short-axis windows, which were not assessed in those studies. Specifically, the parasternal short-axis window is valuable for evaluating the paradoxical movement of the interventricular septum in cases of right ventricular pressure overload and for assessing segmental dysfunctions. This evaluation has also been performed using a transesophageal probe in transthoracic studies,^(4,5)^ providing good-quality images but at a higher cost.

Furthermore, the reported rates of IVC evaluation vary in previous studies (ranging from 33.3% to 92.6% of cases),^(6,9,12)^ reaching 100% only when using the partial deflation technique of the pneumatic mattress^(8)^ because of the difficulty in obtaining the subcostal window in patients ventilated in the prone position. In our study, the IVC was adequately visualized in all evaluated patients.

Consistent with previous studies^(6,7)^ we observed a low rate of PASP estimation using the peak tricuspid regurgitation flow velocity. However, most patients did not present with significant regurgitation in the conventional position, which could be attributed to the fact that the sample consisted of healthy individuals.

The quality of the images acquired in the prone position was satisfactory. Although a statistically significant difference in image quality was observed in the parasternal window (p=0.015), this difference was unlikely to have meaningful clinical implications. The distinction primarily resulted from a shift between "excellent" and "good" image quality scores. Both scores indicate adequate image quality for diagnostic purposes, suggesting that, despite statistical significance, the overall technical feasibility and clinical utility of echocardiography in the prone position remain unaffected.

However, although no statistically significant difference was observed in the quality of images obtained in the apical window, likely due to the small sample size, 50% of the participants evaluated in the prone position had poor or fair quality scores, whereas 80% assessed in the conventional position showed good or excellent quality. These findings suggest a reduction in apical window image quality in the prone position; however, it remains feasible to obtain important variables for the clinical management of patients.

Although a statistically significant difference was observed in the duration of the echocardiographic examination between positions, a 4.2-min difference in the median duration is unlikely to be clinically meaningful. In the studied sample, the longer examination time and the adopted position did not result in any reports of discomfort in patients. However, none of the patients had conditions that might have predisposed them to discomfort in the prone position, such as osteomuscular disorders or breast implants. Furthermore, critically ill patients are often intubated and sedated, which minimizes potential discomfort during image acquisition. These patients typically remain in the prone position for several hours, which further reduces the impact of a slightly longer examination. This modest increase in examination time may be minimized through technical improvements and increased examiner experience.

Quantitatively, no statistically significant differences were observed between the prone and conventional positions for any of the echocardiographic parameters analyzed. These included commonly used variables for hemodynamic evaluation, such as the IVC measurement, which is often used for estimating central venous pressure; the left ventricular outflow tract diameter and VTI of the left ventricular outflow tract, which are widely used for estimating stroke volume and cardiac output; and the E/e’ ratio, which is used to estimate PCWP.

Our study has some limitations. First, the small sample size and inclusion of healthy patients with spontaneous ventilation limit the applicability of the results to patients in other clinical settings. Mechanical ventilation, especially with high positive end-expiratory pressure levels, poses a challenge in echocardiographic assessment owing to alveolar distension, which limits the acoustic window, even in patients in the supine position. Transesophageal echocardiography may be an alternative in cases of limited acoustic windows and has been evaluated in patients with ARDS of other etiologies^(22,23)^ and in COVID-19,^(24,25)^ demonstrating feasibility and safety when performed by experienced operators. However, compared with TTE, transesophageal echocardiography is associated with higher costs, lower availability, greater risk of operator contamination, the need to interrupt enteral feeding, and an increased risk of accidental extubation and aspiration.^(26)^ Therefore, it should be considered a targeted approach for selected cases rather than a first-choice examination for the hemodynamic evaluation of critically ill patients.

Additionally, the average BMI of the patients evaluated in this study was 26 kg/m². In patients with obesity, image acquisition may be compromised by the attenuation of the ultrasound signal due to adipose tissue. Obesity was identified as an important risk factor for requiring mechanical ventilation during the COVID-19 pandemic, and was present in 43% of patients requiring this type of support.^(27)^ Finally, the proposed adaptation was performed on an ultrasound table intended for outpatient examination. Hospital beds, commonly used in intensive care units, have more complex structures with more advanced adjustments for height, inclination, and lateral safety rails. To implement the proposed adaptation in this setting, it is crucial to consult hospital engineering professionals to assess its feasibility and determine the most effective method of implementation.

## CONCLUSION

The use of transthoracic echocardiography in the prone position was feasible following the proposed adaptation, with measurements comparable to those obtained in the conventional position, with good-quality image acquisition, and expansion of the assessed windows. Further studies are needed to validate the accuracy and applicability of this method in real-life scenarios, including critically ill patients in emergency and intensive care units, those receiving mechanical ventilation, and individuals with other comorbidities that may affect the proposed assessment.

## Data Availability

The underlying content is contained within the manuscript.

## References

[1] Alves PR, Gottardo PC (2021). Ultrassonografia à Beira do Leito-O que todo médico deveria saber.

[2] Meyer NJ, Gattinoni L, Calfee CS (2021). Acute respiratory distress syndrome. Lancet.

[3] Ranieri VM, Rubenfeld GD, Thompson BT, Ferguson ND, Caldwell E, Fan E (2012). ARDS Definition Task Force. Acute respiratory distress syndrome: the Berlin Definition. JAMA.

[4] Taha HS, Mohamed AM, Mahrous HA, Shaker MM, Alsayed OS, Sayed HG (2021). Correlation of echocardiographic parameters in prone and supine positions in normal adults using a novel approach. Echocardiography.

[5] Marvaki A, Papachristidis A, Nakou E, Toth E, O’Gallagher K, Fisher R (2020). Innovative transthoracic echocardiographic imaging on prone ventilated patients with COVID-19 using a transesophageal probe. JACC Cardiovasc Imaging.

[6] Ugalde D, Medel JN, Mercado P, Pairumani R, Eisen D, Petruska E (2022). Critical care echocardiography in prone position patients during COVID-19 pandemic: a feasibility study. J Ultrasound.

[7] Cheong I, Otero Castro V, Gómez RA, Merlo PM, Tamagnone FM (2022). Transthoracic echocardiography of patients in prone position ventilation during the COVID-19 pandemic: an observational and retrospective study. Int J Cardiovasc Imaging.

[8] Giustiniano E, Fazzari F, Bragato RM, Curzi M, Cecconi M (2020). Trans-thoracic echocardiography in prone positioning COVID-19 patients: a small case series. SN Compr Clin Med.

[9] Gibson LE, Di Fenza R, Berra L, Bittner EA, Chang MG (2020). Transthoracic echocardiography in prone patients with acute respiratory distress syndrome: a feasibility study. Crit Care Explor.

[10] García-Cruz E, Manzur-Sandoval D, Gopar-Nieto R, Murillo-Ochoa AL, Bejarano-Alva G, Rojas-Velasco G (2020). Transthoracic echocardiography during prone position ventilation: lessons from the COVID-19 pandemic. J Am Coll Emerg Physicians Open.

[11] Santos-Martínez LE, Mendoza-Copa G, García-Cruz E, Álvarez-Álvarez RJ, Bucio-Reta RE, González-Ruiz FJ (2020). Feasibility in the echocardiographic estimation of parameters of the right ventricle in prone position. Arch Cardiol Mex.

[12] Roemer S, Kaminski A, Payne A, Tanel E, Perez Moreno AC, Jaglan A (2020). Feasibility of transthoracic imaging of the heart in the prone position. J Am Soc Echocardiogr.

[13] Del Castillo C, Verdugo F, Appiani F, Yáñez F, Bontá C, Torres-Herrera C (2024). Echocardiogram by apical-subcostal protocol in prone position during invasive mechanical ventilation in cardiovascular intensive care unit. Cardiovasc Ultrasound.

[14] Nuzhny VP, Dernowoy BF, Kibler NA, Prosheva VI, Shmakov DN (2023). Functioning of the Human Heart in the Pron-Position. Kardiologiia.

[15] DuBois D (1916). A formula to estimate the approx-imate surface area if height and weight be known. Arch Intern Med (Chic).

[16] Lang RM, Badano LP, Mor-Avi V, Afilalo J, Armstrong A, Ernande L (2015). Recommendations for cardiac chamber quantification by echocardiography in adults: an update from the American Society of Echocardiography and the European Association of Cardiovascular Imaging. Eur Heart J Cardiovasc Imaging.

[17] Nagueh SF, Middleton KJ, Kopelen HA, Zoghbi WA, Quiñones MA (1997). Doppler tissue imaging: a noninvasive technique for evaluation of left ventricular relaxation and estimation of filling pressures. J Am Coll Cardiol.

[18] Devereux RB, Alonso DR, Lutas EM, Gottlieb GJ, Campo E, Sachs I (1986). Echocardiographic assessment of left ventricular hypertrophy: comparison to necropsy findings. Am J Cardiol.

[19] Morettin PA, Bussab WD (2000). Estatística básica.

[20] Team RDC (2010). R: A language and environment for statistical computing. Computer programme.

[21] Chino JP, Marks LB (2008). Prone positioning causes the heart to be displaced anteriorly within the thorax: implications for breast cancer treatment. Int J Radiat Oncol Biol Phys.

[22] Guarracino F (2011). Transoesophageal echocardiography during prone positioning for ARDS: watching the heart to care for the lung [editorial]. Intensive Care Med.

[23] Mekontso Dessap A, Proost O, Boissier F, Louis B, Roche Campo F, Brochard L (2011). Transesophageal echocardiography in prone position during severe acute respiratory distress syndrome. Intensive Care Med.

[24] Sosa FA, Wehit J, Merlo P, Matarrese A, Tort B, Roberti JE (2023). Transesophageal Echocardiographic Assessment in Patients with Severe Respiratory Distress due to COVID-19 in the Prone Position: A Feasibility Study. Indian J Crit Care Med.

[25] García-Cruz E, Manzur-Sandoval D, Martínez DS, Gopar-Nieto R, Jordán-Ríos A, Díaz-Méndez A (2022). Focused transesophageal echocardiography in critical care: the COVID-19 pandemic. J Cardiovasc Echogr.

[26] Ajam M, Drake M, Ran R, Mukundan S, Masri A, Rahmouni H (2022). Approach to echocardiography in ARDS patients in the prone position: A systematic review. Echocardiography.

[27] Helvaci N, Eyupoglu ND, Karabulut E, Yildiz BO (2021). Prevalence of obesity and its impact on outcome in patients with COVID-19: a systematic review and meta-analysis. Front Endocrinol (Lausanne).

